# Two Methods of AuNPs Synthesis Induce Differential Vascular Effects. The Role of the Endothelial Glycocalyx

**DOI:** 10.3389/fmed.2022.889952

**Published:** 2022-06-29

**Authors:** Daniel Alberto Maldonado-Ortega, Gabriel Martínez-Castañón, Gabriela Palestino, Gabriela Navarro-Tovar, Carmen Gonzalez

**Affiliations:** ^1^Facultad de Ciencias Quimicas, Universidad Autonoma de San Luis Potosi, San Luis Potosi, Mexico; ^2^Facultad de Ciencias, Universidad Autonoma de San Luis Potosi, San Luis Potosi, Mexico; ^3^Centro de Investigacion en Ciencias de la Salud y Biomedicina, Universidad Autonoma de San Luis Potosi, San Luis Potosi, Mexico; ^4^Consejo Nacional de Ciencia y Tecnología, Benito Juarez, Mexico

**Keywords:** gold nanoparticles, aorta, vascular tone, endothelium, glycocalyx, nitric oxide

## Abstract

AuNPs are synthesized through several methods to tune their physicochemical properties. Although AuNPs are considered biocompatible, a change in morphology or properties can modify their biological impact. In this work, AuNPs (~12 to 16 nm) capping with either sodium citrate (CA) or gallic acid (GA) were evaluated in a rat aorta *ex vivo* model, which endothelial inner layer surface is formed by glycocalyx (hyaluronic acid, HA, as the main component), promoting vascular processes, most of them dependent on nitric oxide (NO) production. Results showed that contractile effects were more evident with AuNPsCA, while dilator effects predominated with AuNPsGA. Furthermore, treatments with AuNPsCA and AuNPsGA in the presence or absence of glycocalyx changed the NO levels, differently. This work contributes to understanding the biological effects of AuNPs with different capping agents, as well as the key role that of HA in the vascular effects induced by AuNPs in potential biomedical applications.

**Graphical Abstract:**
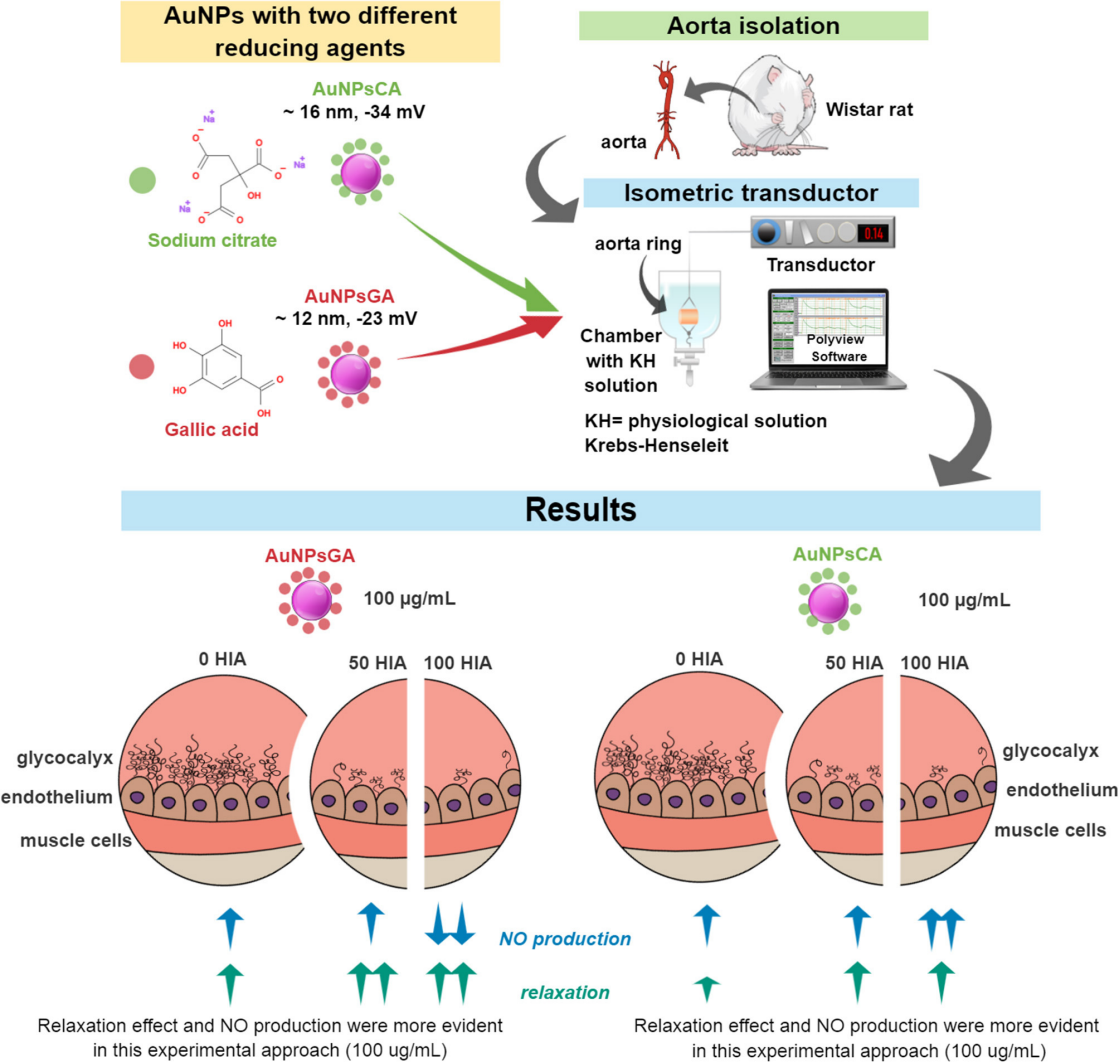
The synthesized AuNPs (~12 to 16 nm) were reduced and capped with two acids: sodium citrate (CA) and gallic acid (GA). These compounds were evaluated in isolated rat aortic rings, an *ex vivo* model, through changes in the vascular tone (vasodilation/vasocontraction) associated with nitric oxide production (NO). The endothelial inner layer surface of the aortic rings is formed by glycocalyx (hyaluronic acid, HA, as the main component), promoting vascular processes, most of them dependent on NO production. The contractile effects were more evident with AuNPsCA, while dilator effects predominated with AuNPsGA. Furthermore, treatments with AuNPsCA and AuNPsGA in the presence or absence of glycocalyx, using hyaluronidase (HIA) 50 or 100 U modified both the magnitude on the vascular tone, as well as the NO levels. The graphical abstract represents the effects related to the concentration of 100 ug/ml of AuNPs, compared to the control and to increasing concentrations of AuNPs with and without enzyme treatment.

## Introduction

To date, the knowledge gained by nanotechnology has offered a plenty variety of nanoparticles (NPs), which have unique properties, such as tunable surface functionality. Gold nanoparticles (AuNPs) are highly remarkable in biomedicine due to their physicochemical, electronic, and intrinsic optical properties (1–4).

AuNPs are widely used in biomedicine as antitumor drug delivery vehicle (5–7), theragnostic platforms (8, 9), thermotherapy (10, 11), gene therapy (12, 13), and diagnostic (14).

AuNPs can be synthesized tuning size and shape particles by different strategies; for instance, thermal, electrochemical, chemical methods, and biosynthesis (15–21). Particularly, chemical methods use a gold salt (Au^3+^) precursor and different reducing agents, such as sodium borohydride, sugars, polyols, gallic acid (GA), and citric acid (CA). Molar proportions of reagents, temperature and reaction time influence the shape and particle size (22).

In this sense, the formation of AuNPs occurs in various steps. Firstly, the dissociation of HAuCl_4_ (strong acid) in water to H^+^ + ${\text{AuCl}}_{4}^{-}$, and CA to citrate (weak conjugated base) + H^+^. Later, citrate molecules provide electrons to the metallic ions, which form seed particles. Then, some AuCl_3_OH^−^ molecules interact with the seed particles to growth into AuNPs (23). Finally, the excess of citrate molecules interacts with the AuNPs surface to increase steric and electrostatic repulsions between particles to increase colloidal stability (24). Similarly, the formation and stabilization of AuNPs occurs in presence if GA. However, this last synthesis is carried out at high pH, favoring the complexation of Au^3+^ by -OH ions and gallate base and consequently, controlling the nucleation process to obtain smaller nanoparticles with homogeneous particle size (25).

The different AuNPs synthesis methods can confer other biological effects. For instance, the spherical AuNPs shape, synthesized with citrate (AuNPsCA; 16–20 nm/150 mM), inhibits cell proliferation in human cholangiocarcinoma cells after 24 h (26). The use of other reduced agents such as GA in spherical AuNPsGA (15 nm/150 μM) reduced the ability to inhibit the growth of cervical cancer cells after 24 h. However, AuNPsGA at the same concentration did not affect normal Vero kidney cells (27).

Interestingly, [Freese et al. (28)] reported that spherical AuNPsCA (18 and 65 nm, 250 μg/mL) can be internalized by human dermal microvascular endothelial cells after 24 h exposure, showing no toxic effects and suggesting that target biology plays a decisive role in toxicity of AuNPsCA (28).

Various spherical sizes of AuNPsCA (3, 5, 7, 10, 30, 60 nm), in concentration of 0.24 to 15.6 μg/mL do not alter the endothelial permeability either promote the release of pro-inflammatory mediators, such as prostaglandins I_2_ and E_2_ in rat brain microvessel endothelial cells. However, the smallest AuNPsCA (3–7 nm) tend to accumulate into these cells. Moreover, AuNPsCA of 3 nm (7.8 μg/mL or higher) show a moderate decline of the viability cell but unmodified the morphology after 24 h exposure (29).

Pan et al. (30) showed that the cytotoxicity induced by spherical AuNPsCA (1–2 and 5 nm; 110 μM, 24 h of exposure) is size-dependent in evaluations performed in cancer cell cultures of SK-Mel-20 human melanoma, HeLa human cervix carcinoma, L929 mouse fibroblast and J774A1 cells after 24 h of exposure (30), meanwhile, Chi-Ming et al. (31), showed that AuNPs in the range of 3–5 nm after 30 min of exposure, suppressed the vascular endothelial growth factor (VEGF)-induced activation of Akt/eNOs signaling pathway in rhesus macaque choroid-retinal endothelial cell line RF/6A derived from the choroid-retina with no signs of cytotoxicity.

Moreover, 10 nm spherical AuNPsCA were exposed to the NO donor ruthenium complex Cis-[Ru(bpy)_2_(NO)(4PySH)]. (PF_6_)_3_ in a range concentration of 0.3 nM to 10 μM, the combination induced a vasodilator effect from the concentration of 5 μM in precontracted isolated rat aortic rings (32). Recently, 20 nm AuNPsCA promoted a transient vasodilation in mouse 4T1 tumors after intragastric and intravenous administration of these NPs. This effect could be mediated at least in part by the NO production and did not accelerate the tumor growth (33).

On the other hand, a study with spherical AuNPs (14 nm, 1 μg/mL) synthesized with eggplant extract and coated with HA evaluated the incorporation of a small-interfering ribonucleic acid-specific (to silence the expression of IAP-2, an inhibitor of apoptosis). The results showed that the modified AuNPs decreased the cell proliferation and triggered pronounced cell apoptosis in A456 human lung carcinoma cells after 48 h of exposure (34). Also, spherical AuNPs of 30 nm synthesized with eggplant extract coated with HA and metformin 4 μg/mL, induced a reduction in G2/M phase and molecular level apoptosis in HePG2 human liver cancer cells after 48 h exposure, while for that free metformin ranged from 10 μg/mL (35).

The HA is a macromolecule that makes up the endothelial glycocalyx (among other glycosaminoglycans and proteoglycans), which is produced by endothelial cells (36, 37). HA has been implicated in NO production when endothelial cells are exposed to wall shear stress (37, 38). NO is an essential mediator in the regulation of vascular tone since it promotes muscle relaxation and is synthesized from L-arginine by activation of different NO synthase (NOS) isoforms, endothelial NOS (eNOS), neuronal NOS (nNOS), and inducible NOS (iNOS) (39, 40).

In this context, the glycocalyx can potentially mediate mechanical transduction; since when the glycocalyx layer is removed, flow-dependent vasodilation and NO production is altered (36, 41). For example, in an experiment where 4–6 cm length of the rat's right superficial femoral artery was incubated with 14 μg/mL of hyaluronidase (HIA) for 20 min to remove HA, NO levels decreased as well as the vasodilation (42).

Our research group has shown that other metallic nanoparticles, such as silver nanoparticles can modulate smooth muscle contraction (43–45) and moreover the AuNPsGA promoted a transient smooth muscle contraction in precontracted rat isolated tracheal rings (46). However, studies on the actions of the AuNPs and their interaction with structures of blood vessels are poorly studied. Thereby, we aim to evaluate the participation of glycocalyx structures in vascular actions induced by AuNPs synthesized by two different methods.

## Materials and Methods

### Chemicals

HAuCl_4_, acetylcholine (ACh), Phenylephrine (Phe), vanadium (III) chloride, N-(1-naphthyl) ethylenediamine dihydrochloride (NEED), sulfanilamide (SULF), bovine serum album (BSA), HIA (Type IV-S: from bovine testes), glutaraldehyde, ethanol (>98%), NaCl, KCl, KH_2_PO_4_, MgSO_4_, CaCl_2_, C_8_H_18_N_2_O_4_S 4-(2-hydroxyethyl)-1-piper-azineethanesulfonic acid (HEPES) were purchased from Sigma Chemical Company (St. Louis MO, USA).

### Synthesis of AuNPsGA and AuNPsCA

AuNPsGA were synthesized as described by Moreno-Alvarez et al. (47). Briefly, 10 mL of deionized water containing 0.001 mol of GA were added, under magnetic stirring, to 100 mL of a 0.001 M gold (III) solution prepared from a stock solution made with HAuCl_4_ salt and deionized water. Then the pH value was adjusted to 10 using a 1.0 M NaOH solution (30 min). This reaction time was determined using UV-Vis spectroscopy (S2000-UV-Vis fiber) following the development of the AuNPs plasmon. A decrease in the intensity of the signal at 262 nm confirmed the adsorption of the GA to AuNPs (47).

On the other hand, AuNPsCA were synthesized by Turkevich method (48) using 40 mL of a 0.001 M gold (III) solution prepared from a stock solution with HAuCl_4_ salt and deionized water at a temperature of 90 °C were added, under magnetic stirring, to 4 mL of deionized water containing 0.0002 mol of CA. The reaction was kept for 30 min (monitoring by UV-Vis spectroscopy). The decrease of intensity of the signal at 340 nm confirmed the adsorption of the CA to AuNPs.

### Transmission Electron Microscopy Analysis

Physical characterization of synthesized AuNPs was performed by transmission electron microscopy (TEM) using JEM−1230 (JEOL company, Peabody, MA) instrument working at an accelerating voltage of 100 kV. The AuNPs were analyzed after suspension in water and subsequent deposition onto carbon-coated grids. Images obtained were used to determine the mean size and standard deviation of particle sizes by measuring over 100 particles in random fields of view. Collected data were analyzed by ImageJ software (Version 1.50, National Institutes of Health, Bethesda MD, USA).

### Dynamic Light Scattering Analysis

The hydrodynamic diameter and zeta potential of AuNPs were determined by dynamic light scattering (DLS) in a Beckman Coulter zeta potential and submicron particle size analyzer DelsaNano C. Measurements were performed by number distribution in a normalized scale. Measurements were conducted at 25 C using water as dispersant medium (viscosity 0.8872 cP; dispersant dielectric constant 78.5; dispersant refraction index 1.330). Both AuNPsGA and AuNPsCA stocks (2 mg/mL) were diluted 1:1 v/v to perform DLS analysis.

### Dispersion of AuNPs

AuNPs were suspended in sterile deionized water at 3.5 mg/mL and dispersed by sonication (10 min) at ambient temperature using a Cole-Parmer 470 50 W ultrasonic tip processor at 45 kHz of frequency.

### Tissue Preparation

Adult male Wistar rats (300–350 g) were sacrificed by overdose injection of sodium pentobarbital under animal protocols approved by the Animal Care and Use Committee of the Universidad Autonoma de San Luis Potosi (CEID2014033, CEID202003). The experiments were performed as previously described (45).

The aorta was excised, cleaned of adherent tissue, and cut into 3–4 mm length segments. Then, individual rings with endothelium were suspended in organ baths containing buffered Krebs-Henseleit (KH) solution (118 mM NaCl, 4.6 mM KCl, 1.2 mM KH_2_PO_4_, 1.2 mM MgSO_4_, 1.75 mM CaCl_2_, 20 mM HEPES) free of pharmacological blockers, and with different either 50 U HIA or 100 U HIA for 20 min and then washed out with KH solution containing 1 % BSA for 10 min to remove of HIA. The solutions were kept at 37°C and pH of 7.4.

### Vascular Tone of Rat Aortic Rings

The aortic rings with or without HIA were suspended from a Radnoti isometric transducer in organ baths containing buffered KH solution. A passive load of 2 g was applied, and the aortic segments were allowed to equilibrate for an hour. Rat aortic vessels were precontracted with 2 μM of Phe, followed by AuNPs exposure (0.1, 1, 10, and 100 μg/mL). The solution was kept at 37°C and pH of 7.4. Isometric changes in tension were monitored using Polyview software (Astro-Med, Inc. Grass Instrument Division). Quantitative evaluation of the effects was measured as the percentage of reduction in the vascular tone induced by the AuNPs sample, to the 100% of contraction triggered by Phe. The magnitudes were normalized with Image J software (National Institute of Health, Bethesda, MD).

### Nitric Oxide Production

NO production was quantified indirectly by measuring nitrites (NO_2_) and nitrates (NO_3_), which represent the NO metabolism final products, using the Griess method (44). Briefly, 100 μL aliquots of KH solution contacting with aortic rings with or without HIA treatment or AuNPsGA and AuNPsCA were sampled into 96 well plates and incubated for 30 min in the presence of 10 μL of NEDD (0.1%, w/v), 10 μL of SULF (2%, w/v) and 80 μL of vanadium (III) chloride (50 mM) at 37°C. After incubation, the absorbance of each sample was measured with plate iMark^TM^ microplate reader (BIO-RAD, serial number 10923), with an emission filter set at 560 nm. NO_2_/NO_3_ concentration was calculated using NO_2_ standard curve.

### Statistical Analysis

Data were collected from three independent experiments. After confirming normal distribution by the Kolmogorov-Smirnov's test, a one-way analysis of variance (ANOVA) or two-way ANOVA (Factorial design) followed by Fisher's Least Significant Differences test to detect significant variations among treatments. Statistical analysis was performed using the Statistica 10 software package (StatSoft, Tulsa, OK, USA), whereas Graph Pad Prims V 5.01 (Graph-Pad Software Inc.) was used for data plotting. Statistical significance for all analyses was accepted at *P* < 0.05.

## Results

### AuNPs Morphology and Surface Charge

**Figure 1:**
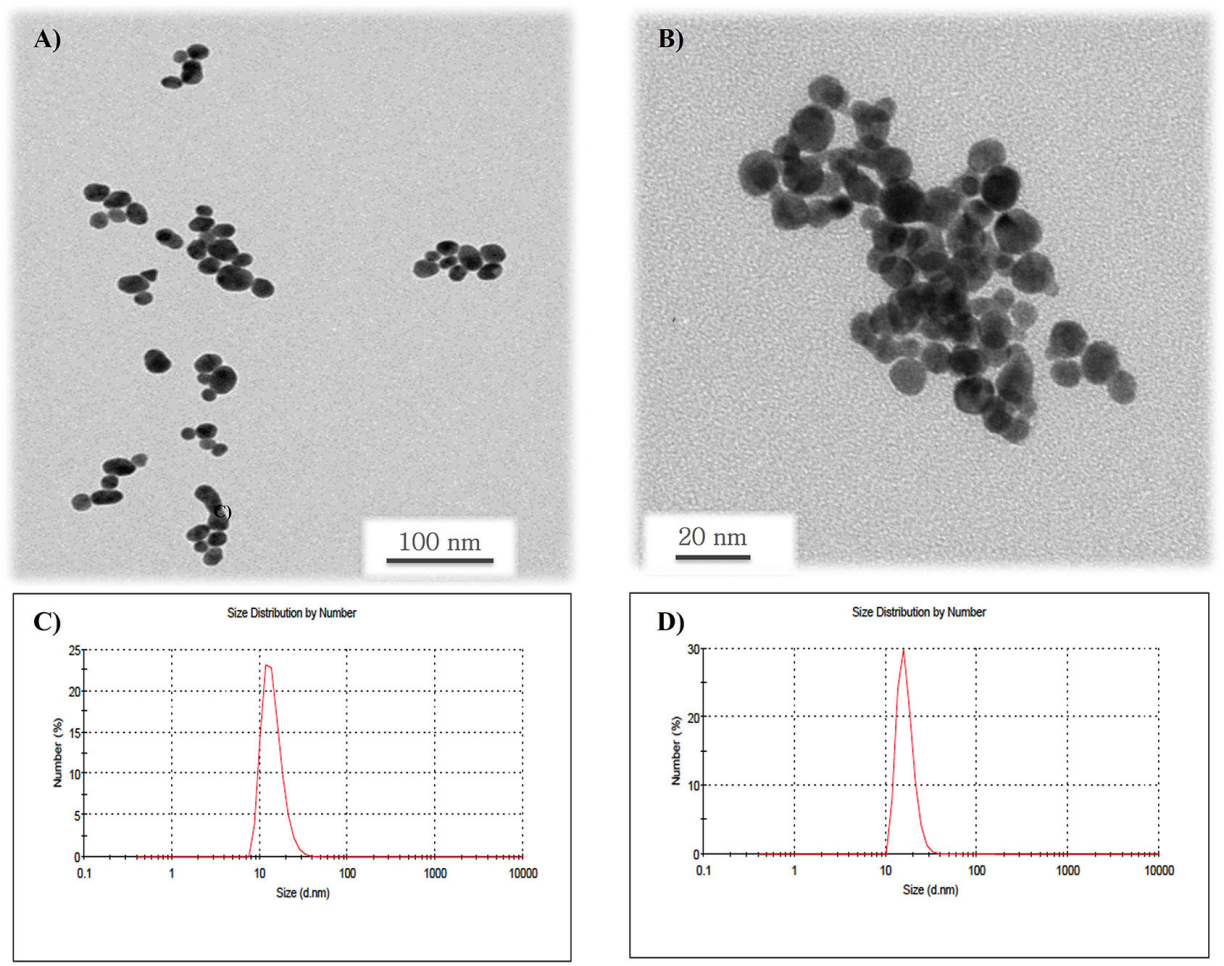
TEM micrographs of AuNPs. **(A)** Image shows the spherical shape of the AuNPsGA dispersed in water with a mean particle size of 11.6 nm ± 2.82; **(B)** image shows spherical AuNPsCA with a mean particle size of 15.8 nm ± 3.56; **(C)** histogram shows the DLS size distribution of the AuNPsGA with a peak value of 14.4 nm; and **(D)** histogram shows the DLS size distribution of the AuNPsCA with a peak value of 16.5 nm.

TEM analysis revealed that AuNPsGA (Figure 1A) and AuNPCA (Figure 1B) have spherical shape and a size distribution with a mean particle size of 11.6 nm ± 2.82 and 15.8 nm ± 3.56, respectively. DLS analysis determined for AuNPsGA a range size from 7.9 to 37 nm, with a peak of 14.14 nm (Figure 1C) and a mean surface charge of−23.5 mV. On the other hand, AuNPsCA showed a range size from 10 to 30 nm with a peak of 16.56 nm (Figure 1D) and a mean surface charge of −34.5 mV (Table 1).

**Table 1:** Comparative parameters between TEM and DLS.

**Nanoparticle**	**Particle size (nm), TEM**	**Hydrodynamic diameter (nm), DLS/Peak (nm)**	**Zeta potential (mV), pZ**	**Polydispersion index (PDI)**
AuNPsCA	15.8 ± 3.56	10 to 30 / 16.56	−34.5 ± 6.47	0.369
AuNPsGA	11.6 ± 2.82	7.9 to 37/ 14.14	−23.5 ± 6.61	0.188

### AuNPs Modulate the Vascular Tone

**Figure 2:**
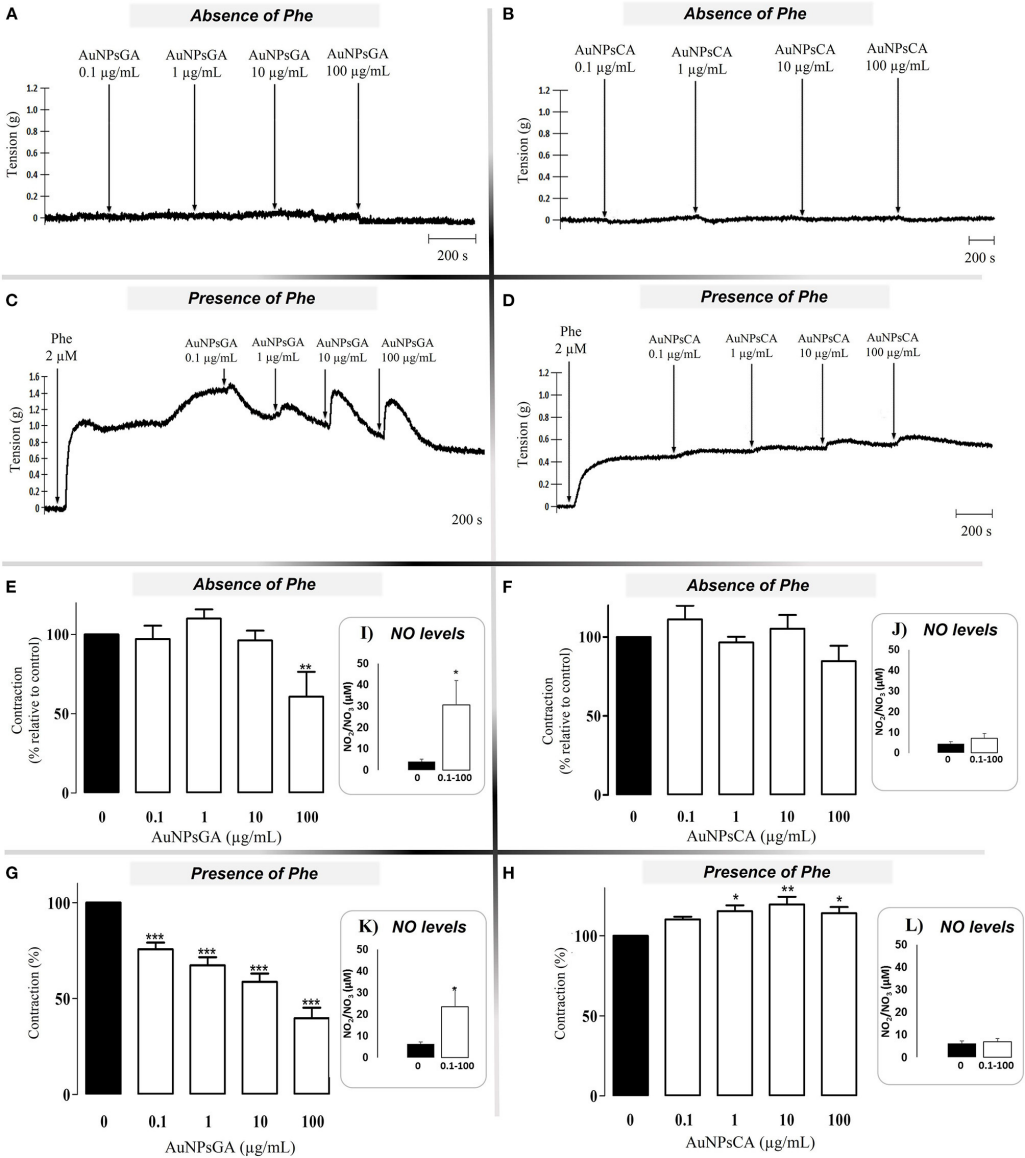
Effects induced by increasing concentrations of AuNPsGA and AuNPsCA. Cumulative concentrations (μg/mL) of AuNPs of AuNPsGA **(A)** and AuNPsCA **(B)** did not induce an effect on vascular tone in the aortic rings in absence of Phe 2 μM. Cumulative doses of AuNPsGA **(C)** and AuNPsCA **(D)** induced a vasodilator and vasocontraction effect on precontracted aortic rings with Phe 2 μM, respectively. Results are representative of three independent experiments. Percentage of contraction induced by AuNPsGA and AuNPsCA (0.1, 1, 10, and 100 μg/mL) in the absence **(E,F)** and presence **(G,H)** of Phe 2 μM, calculated as the percentage of tension based on 100% contraction induced by Phe 2 μM. NO production, in the absence and presence of AuNPsGA and AuNPsCA (0.1, 1, 10 and 100 μg/mL), also in the absence **(I,J)** and presence **(K,L)** of Phe 2 μM was determined by Griess method. Values are represented as mean ± SEM (*n* = 3). *P < 0.05, **P < 0.01 and ***P < 0.001 vs. control (0 μg/mL).

Non-precontracted aortic rings were treated with increasing concentrations of AuNPsGA and AuNPsCA (0.1–100 μg/mL). The concentrations were directly administered into the organ baths containing the aortic rings. Data show that the cumulative concentration of 100 μg/mL AuNPsGA did not modify the vascular basal tone (Figures 2A,E). Cumulative concentrations of AuNPsCA did not alter the vascular basal tone (Figures 2B,F). However, the aortic rings were precontracted with Phe 2 μM; AuNPsGA induced vasodilation at all administrated cumulative concentrations (0.1–100 μg/mL) (Figures 2C,G), and in a different fashion than the non-precontracted rings. In contrast, AuNPsCA induced a contractile effect, being significant since the concentration of 1 μg/mL (Figures 2D,H).

**Figure 3:**
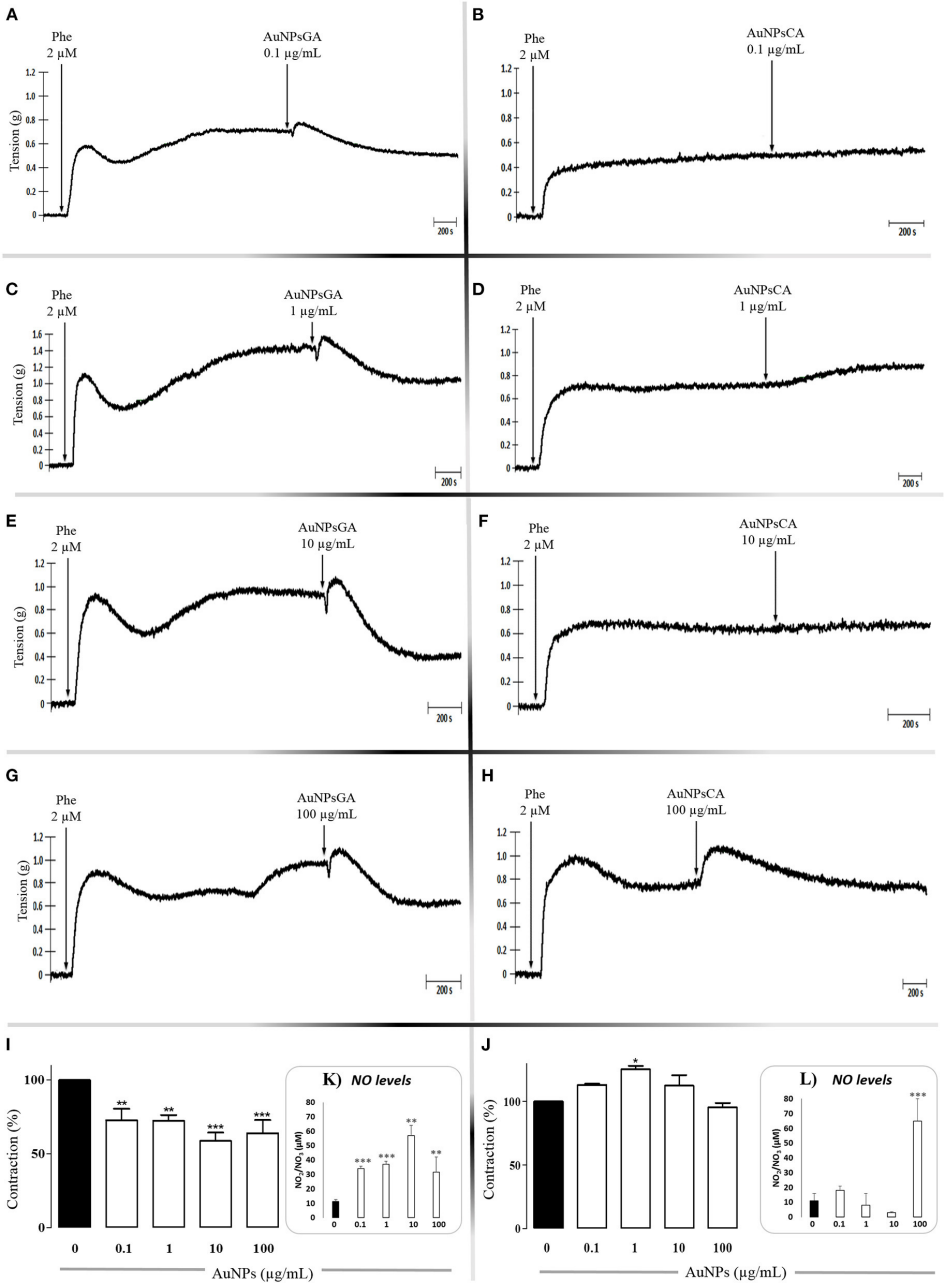
Effects induced by singles concentrations of AuNPsGA and AuNPsCA. Effects induced by single concentrations of AuNPsGA **(A)** 0.1, **(C)** 1, **(E)** 10 and **(G)** 100 μg/mL; and AuNPsCA **(B)** 0.1, **(D)** 1, **(F)** 10, **(H)** 100 μg/mL on pre-contracted aortic rings with Phe 2 μM. Results are representative of three independent experiments. Percentage of contraction induced by AuNPsGA (0.1, 1, 10, and 100 μg/mL) **(I)** AuNPs-CA (0.1, 1, 10, and 100 μg/mL) **(J)** on pre-contracted aortic rings with Phe 2 μM, calculated as the percentage of tension based on 100% contraction induced by Phe 2 μM. NO production, in absence and presence of AuNPsGA (0.1, 1, 10, and 100 μg/mL) **(K)** and AuNPsCA (0.1, 1, 10, and 100 μg/mL) **(L)** was determined by Griess method. Values are representing as mean ± SEM (*n* = 3). *P < 0.05, **P < 0.01 and ***P < 0.001 vs. control (0 μg/mL).

To infer responsible concentration(s) of AuNPsGA and AuNPsCA in modulating the vascular tone, AuNPsGA and AuNPsCA were administrated in single concentrations, using precontracted rings with Phe 2 μM. AuNPsGA induced vasodilation at all single concentrations used (0.1–100 μg/mL); (Figures 3A,C,E,G,I), while AuNPsCA (0.1–100 μg/mL) keeps or increases the vasoconstriction displayed by the Phe (Figures 3B,D,F,H).

Data suggest that the physiological effect exerted by AuNPs depends on both the synthesis method and the particle concentration. Thus, more studies were performed to figure out the role of glycocalyx, since it is the first contact of AuNPs with the cell.

### AuNPs Physiological Effects Are Dependent on Glycocalyx

HA indirectly regulates the vascular tone since its removal decreased the NO levels (38). We performed a HIA enzyme treatment to aortic rings to remove HA before exposing it to AuNPsGA and AuNPsCA. Then, compare each event in the presence of the enzyme to their control (no enzyme). 50 U HIA treatment did not modify the vasodilator effect induced by ACh (See Supplementary Figure 1A). However, 100 U HIA treatment, the vasodilation promoted by ACh was reduced ~ 10 % vs. the treatment with HIA 50 U and ~ 50 % over the effect induced by ACh (HIA 0U) (Supplementary Figures 1B–D).

**Figure 4:**
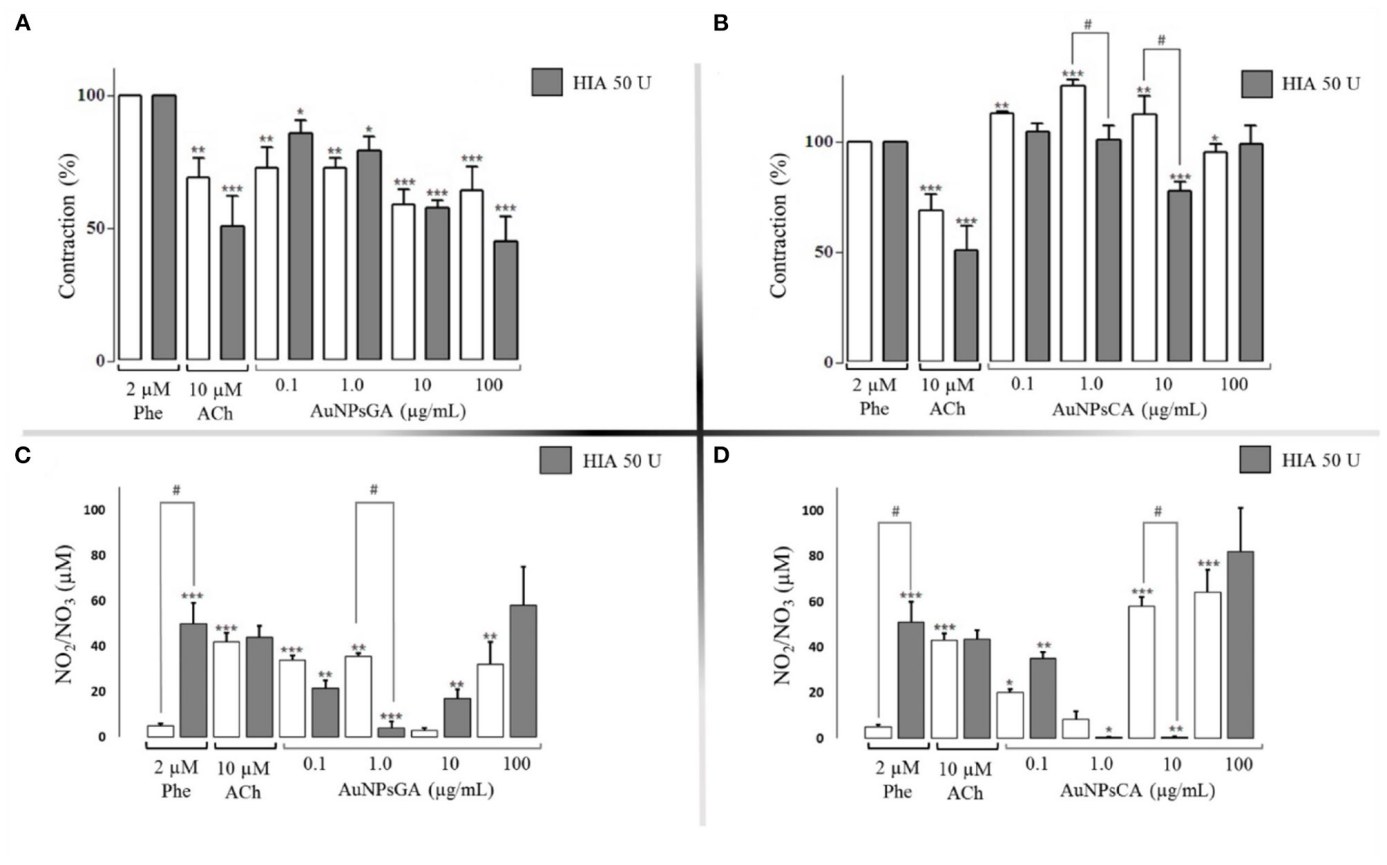
Effects induced by AuNPsGA and AuNPsCA previously treatment with HIA 50 U. Percentage of contraction induced by single concentrations of AuNPsGA **(A)** 0.1, 1.0, 10, and 100 μg/mL, and **(B)** singles concentrations of AuNPsCA **(A)** 0.1, 1.0, 10, and 100 μg/mL. Graphics A and B show aortic ring contraction with previous treatment with either 0 U HIA (white bars) or 50 U HIA (gray bars) on precontracted aortic rings with Phe 2 μM. The contraction was calculated as the percentage of tension based on 100% contraction induced by Phe 2 μM. Results are representative of three independent experiments. NO production, in the presence of AuNPsGA **(C)** and AuNPsCA **(D)** (0.1, 1, 10, and 100 μg/mL), also with previous treatment with either 0 U of HIA (white bars) or 50 U HIA (gray bars). NO levels were determined by Griess method. Values are represented as mean ± SEM (*n* = 3). *P < 0.05, **P < 0.01 and ***P < 0.001 vs. control with Phe (white bars) or the control with Phe and 50 U HIA (gray bars). ^#^*P* < 0.001 differences between no presence of HIA 50 U and presence of HIA 50 U.

The treatment with 50 U HIA enhanced the vasodilator effect of AuNPsGA 100 μg/mL in aortic rings compared to those concentrations in the absence of HIA (Figure 4A). In contrast, AuNPsCA (0.1 and 1 μg/mL) administrated in the aortic rings with the same enzymatic treatment restored the Phe contractile effect, which increased in the absence of HIA 50 U, suggesting the important role of HA, as part of the endothelial glycocalyx on the effect induced by AuNPs (Figure 4B). However, AuNPsCA 100 μg/mL in the presence of HIA did not change the contractile effect vs. the absence of HIA. The physiological effects of AuNPsGA and AuNPsCA at single doses when aortic rings were previously treated with 50 U HIA, are shown in Figure 4.

**Figure 5:**
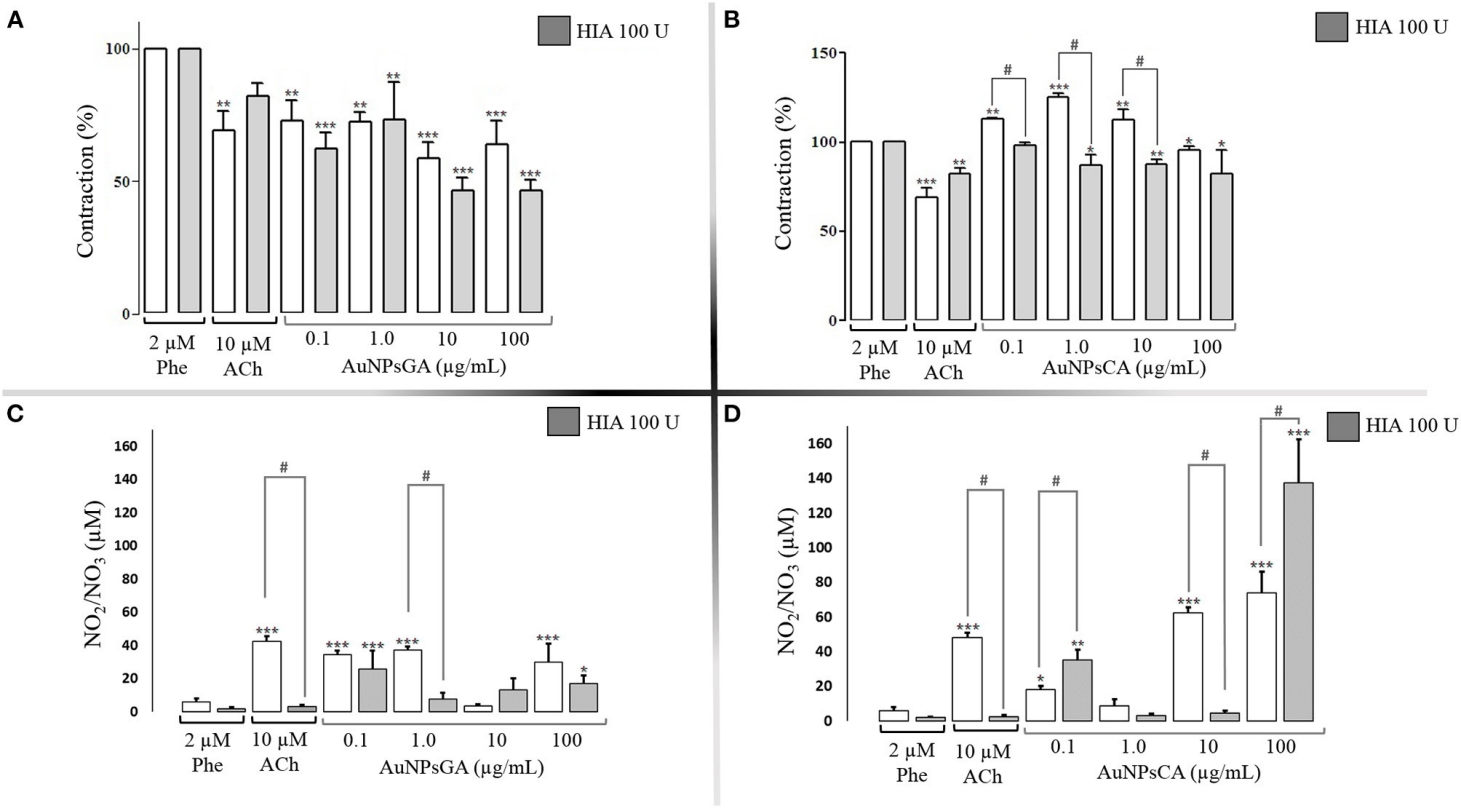
Effects induced by AuNPsGA and AuNPsCA previously treatment with HIA 100 U. Percentage of contraction induced by single concentrations of AuNPsGA **(A)** 0.1, 1.0, 10 and 100 μg/mL, and **(B)** singles doses of AuNPsCA **(A)** 0.1, 1.0, 10, and 100 μg/mL. Graphics A and B show aortic ring contraction with previous treatment with either 0 U HIA (white bars) or 100 U HIA (gray bars) on precontracted aortic rings with Phe 2 μM. The contraction was calculated as the percentage of tension based on 100% contraction induced by Phe 2 μM. Results are representative of three independent experiments. NO production, in the presence of AuNPsGA **(C)** and AuNPsCA **(D)** (0.1, 1, 10, and 100 μg/mL), also with previous treatment with either 0 U of HIA (white bars) or 100 U HIA (gray bars). NO levels were determined by Griess method. Values are represented as mean ± SEM (*n* = 3). *P < 0.05, **P < 0.01 and ***P < 0.001 vs. control with Phe (white bars) or the control with Phe and 50 U HIA (gray bars). ^#^*P* < 0.001 differences between no presence of HIA 50 U and presence of HIA 50 U.

On the other hand, the treatment with 100 U HIA enhanced the vasodilator effect of AuNPsGA at the concentration of 0.1, 10, and 100 μg/mL (Figure 5A). In contrast, the treatment of AuNPsCA in the aortic rings with the same enzymatic treatment reduced the contraction induced by AuNPsCA (0.1, 10, 10 μg/mL) in comparison to the absence of HIA, suggesting that AuNPsCA modulate the vascular effects through the presence of HA (Figure 5B) as part of the endothelial glycocalyx.

### AuNPs Vascular Effects and Nitric Oxide Production

NO is an endothelium-dependent vasodilator of the smooth muscle, which plays a pivotal role in maintaining homeostatic conditions of the blood vessels. NO under physiological conditions is synthesized by the constitutively expresses enzymes eNOS and nNOS (39). Under the altered situation, inflammation promotes the inducible isoform iNOS generating a higher NO concentration (44).

In this study, the NO production was determined in the KH solution before and after the aortic ring was exposed or not to HIA 50 U/HIA 100 U and treated or not with AuNPsGA and AuNPsCA. The cumulative concentrations of AuNPsGA (Figure 2I) and AuNPsCA (Figure 2J), using the non-precontracted rings, induced NO production release since the AuNPsGA promoted a significant increment vs. the control, six times the control level, but not the AuNPsCA. However, the precontracted rings with Phe 2μM displayed a significant NO production increase induced by AuNPsGA (Figure 2K) about five times vs. control, but not AuNPsCA (Figure 2L). Moreover, the precontracted rings enhanced the NO levels about 1.3 times in comparison with non-precontracted conditions. The addition of single doses of AuNPsGA (Figure 3K) and AuNPsCA (Figure 3L) (0.1, 1,10, and 100 μg/mL) μg/mL induced differential NO levels.

The AuNPsGA treatment with single doses promoted significant production of NO vs. the control, which was associated with the relaxation induced by all the concentrations under study (Figures 3A–K). However, in the AuNPsCA, only the concentration of 100 μg/mL yielded higher production of NO, which could be related at least in part, with a modest relaxation (Figures 3H–L).

Thus, the effect provoked by AuNPsGA and AuNPsCA on the vascular tone is associated with NO, but in a differential production that is in the function of the increasing concentrations of AuNPsGA and AuNPsCA and strongly suggests that could be other(s) mediator(s) induced by AuNPs implicated in the modulation of vascular effects.

When HA was removed by HIA treatment in aortic rings, and later exposed to AuNPsGA and AuNPsCA, variations in the NO production were seen compared to their respective control in the absence of HIA.

In this context, the aortic rings previously exposed to HIA (50 U) in the presence of single concentrations of AuNPsGA of 0.1 and 1.0 μg/mL decreased NO levels in a pattern dependent on the AuNPsGA concentration. However, the concentrations of 10 and 100 μg/mL were associated with a significant vascular relaxation (Figure 4C).

In contrast, AuNPsCA exposed to HIA (50 U) at a concentration of 0.1μg/mL increased the NO levels, and 1 and 10 μg/mL AuNPsCA decreased the NO production (Figure 4D). At the concentration of 100 μg/mL, the NO stimulation was not changed in comparison to the treatment in the absence of HIA 50 U treatment. These fluctuant productions of NO appear not to be related to the effect induced by AuNPsCA upon the vascular tone, suggesting that other mediators or structures HA-dependent are involved in this physiological profile.

Whereas, the aortic rings previously exposed to HIA (100 U) in the presence of single concentrations of AuNPsGA showed that at a concentration of 1 μg/mL decreased the NO production and it was not associated with the contractile effect (Figures 5A,C), meanwhile, the concentration of 10 and 100 μg/mL, even change the levels of NO vs. the correspondent treatment with HIA increasing the vasodilation (Figures 5A,C). In the case of aortic rings previously exposed to HIA 100 U and treated with the AuNPsCA, the fluctuation on the NO production was contrasting and associated with the vascular tone at the different AuNPs concentrations evaluated (Figures 5B,D), which biological pattern was like those displayed with the HIA 50 U treatment.

In this study, we found that part of the physiological effects depended on the AuNPsGA/AuNPsCA concentration and the endothelial glycocalyx HA destined to regulate the vascular tone. The mediator detected was the NO, that plays a pivotal role in the vascular effects of AuNPsCA associated to the glycocalyx, but other mechanism can be associated.

## Discussion

It is undeniable that studies of AuNPs are supplying relevant information for their biomedical applications. However, the biological mechanisms involved are not fully described in the literature. Considering the leading site of AuNPs distribution is blood circulation, most of the toxicological evaluations of AuNPs in animals have been conducted by intravenous administration of AuNPs (4, 7). The vascular system is divided into (1) heart, as a central pump focuses on distributing blood to (2) major vessels, which delivers and returns blood from the heart to (3) minor vessels, which are finally distributed in the organs and tissues (49). Both major and minor vessels have an inner endothelial cell layer (endothelium). Together, the muscle cells and endothelial cells regulate the vascular tone through vasoactive factors (50, 51). One of these factors is nitric oxide (NO), which is synthesized by the nitric oxide synthase (NOS) (39, 40).

The vascular effects induced by NO are controversial, including the regulation of the vascular tone, which are in function of a list of factors such as: (a) the presence of O2.-, this kind of factor can modify the half time, bioavailability, and the concentration of NO, for instance the NO varies in function of the oxygen tension and the O2.-. The concentration range of 10-50 nM, NO has a half-life time around 3-5 s, in excessive concentration of 300 nM, the half-life time could be longer than 30 s; (b) the presence of scavengers like oxyhemoglobin to yield methemoglobin and inorganic nitrate; (c) the reaction of NO with thiols groups presents in the proteins, which formed the S-nitroso thiols; (d) The biological location of the NOS isoforms in conditions by the normal physiological situation, which can produce a balance of NO concentration in the order or picomolar/nanomolar (eNOS/nNOS), or in altered conditions produces NO in the order or micromolar (iNOS). For example, the high NO production that characterized iNOS isoform is expressed in the vascular smooth muscle cells following exposure to pro-inflammatory cytokines promote; hypotension, cardiodepression and vascular hyporeactivity in septic shock (40, 52, 60).

In the vascular smooth muscle cells of thoracic aorta from rat, 5 nm AuNPs (100 mM) synthesized with sodium ascorbate induced vasodilatation dependent on the NP concentration and endothelium-independent by the ability to activate sensitive potassium channels calcium (53). Our findings demonstrated induction of the vasodilation only with AuNPsGA, while AuNPsCA showed contractile effects in aortic rings. A preliminary analysis showed that AuNPsCA could be deposited onto the tissue and then induce contractile effects (Supplementary Figure 2).

An earlier report with AuNPsCA coupled to NO donors induced a dilatory effect on rat aortic rings (32). In our work, the role of NO on the AuNPs vasodilator and vasoconstriction effects were evaluated; AuNPsGA and AuNPsCA actions were related to NO production. The variation on NO levels suggests the activation of different isoforms of NOS, which leads to vasodilation and vasoconstriction, saw that AuNPsGA concentrations increased the basal NO production inducing vasodilation. Furthermore, 0.1 to 10 μg/mL of AuNPs-CA did not increase the basal NO production and caused vasoconstriction, but the AuNPsCA at 100 μg/mL induced NO production with no vasoconstriction effect may explain these results by activating various signaling pathways or the AuNPsCA interaction with the endothelial surface structures. For instance, [Santos et al. (54)] compared the vasorelaxation induced by AuNPs capped with either thioglycolic acid (AuNPTGA) or thioglycolic acid modified with berberine (AuNPTGA-BS). AuNPTGA did not induce vasorelaxation, but the incorporation of berberine onto the particle surface triggered vasorelaxation by cytosolic calcium ions concentration decreased (54). Mohamed et al. (55) reported a vasodilation effect in isolated aorta rings (male Wistar rats) when exposed to AuNPs with different chemical surfaces. The percentage of relaxation was associated with the chemical surface. At 0.030 M, AuNPsCA induced relaxation of ~70%, while AuNPs with polyvinylpyrrolidone (PVP) and mercaptopolyethylene glycol (mPEG) at the same concentration generated ~60 and ~50%, respectively. The authors reported that citrate capped particles did not alter endothelial-dependent vasodilation previously induced by ACh but attenuated endothelial-independent responses induced by sodium nitroprusside. The capping with PVP attenuated the ACh-induced relaxation, whereas mPEG did not (55).

On the other hand, the slight differences in particle size and the apparent value dispersion could trigger different cellular mechanisms. The synthesis of AuNPs with gallic acid allows a more controlled nucleation of Au due to both gallic acid and -OH ions (pH 10) complexing Au^3+^, resulting in smaller nanoparticles (25) with apparent better dispersion than AuNPsCA, as characterization presents (Table 1). Thus, AuNPsGA (11.6 could ± 2.82 nm) may be more compatible than AuNPsCA with higher diameter (15.8 ± 3.56 nm), and which induced contractions in smooth muscle due accumulation and aggregation of particles (Supplementary Figure 2).

However, the evidence found in this work regarding the glycocalyx's role, particularly from hyaluronic acid (HA), suggest that functional groups and/or chemical conformation of the capping agent exhibit variations in the affinity with HA.

With respect to glycocalyx role, a report by Kumagai et al. (42) in the superficial femoral artery of porcine showed that HIA 15 mU/mL (2 h 37°C) decreased about 50% the NO level (concerning a control with no enzyme) and about 15% vasodilation effect (concerning same control). Thus, alterations of glycocalyx may modify intracellular and cytoskeletal structures and activate NO synthase (eNOS), associated with a low NO production (56). It has been showed that citrate-coated iron oxide NP show strong interaction with proteoglycans and glycosaminoglycans in THP-1 monocytes (57); and particularly, AuNPsCA exhibit high affinity to HA in physiological-like solutions (58). The glycocalyx can entrap and accumulate AuNPs and take part in the receptor-mediated endocytosis (59). In the present work, when aortic rings were treated with HIA and AuNPs, the NO production depended on the concentration of the enzyme concentration being more evident, the physiological effects and NO production induced by AgNPsGA at the concentration of 100 μg/mL in the presence of HIA 50 U, which increased the aortic relaxation associated with NO production; however, 100 U of HIA instead promoted the vascular relaxation, did not change the NO levels at the concentrations of AuNPsGA 100 μg/mL, suggesting that other endogenous agents HA-dependent could modulate the relaxation.

In contrast, AgNPsCA has a different profile upon the physiological effect and NO levels with respect to those showed by AgNPsGA. Our results suggest that AuNPs effects are dependent on the capping agents, and endothelial glycocalyx plays an important role in these actions.

Further details are underway to investigate the interaction of AgNPsCA and AgNPsGA with glycocalyx and the physiological effects under normal and pathological conditions.

## Conclusion

This work shows at the vascular level the effects of AuNPs with two different capping agents. Contractile effects induced by AuNPs may be due to an interaction between AuNPs and the endothelial glycocalyx. Notably, the removal of HA led to NO production modifications, which triggered an intracellular signal for either vasodilation or vasoconstriction effects. The data generated show the biological importance of the reduce/stabilizing agents (GA and CA) used in the chemical synthesis of AuNPs and turn settings applications of NMs in the biomedical area, bioaccumulation, and route of administration. Our findings contribute to understand the AuNPs effects, their mechanism of action in the vascular system and to benefit biosafety of AuNPs.

## Data Availability Statement

The raw data supporting the conclusions of this article will be made available by the authors, without undue reservation.

## Ethics Statement

The animal study protocols CEID2014033 and CEID202003 were reviewed and approved by CONBIOETICA-24-CEI-003-20190726.

## Author Contributions

DM-O: investigation, visualization, writing—original draft, and data curation. GN-T: writing—review and editing, investigation, visualization, and data curation. GM-C and GP: validation and data curation. CG: conceptualization, visualization, writing—original draft, supervision, funding acquisition, project administration, and investigation. All authors contributed to the article and approved the submitted version.

## Funding

This work was supported by Consejo Nacional de Ciencia y Tecnologia through the fellowship of DM-O (633022), CI6-PIFI-09-08-08, Problemas Nacionales CONACyT PN-2017-01-4710.

## Conflict of Interest

The authors declare that the research was conducted in the absence of any commercial or financial relationships that could be construed as a potential conflict of interest.

## Publisher's Note

All claims expressed in this article are solely those of the authors and do not necessarily represent those of their affiliated organizations, or those of the publisher, the editors and the reviewers. Any product that may be evaluated in this article, or claim that may be made by its manufacturer, is not guaranteed or endorsed by the publisher.
