# Supplementary material for: Two Methods of AuNPs Synthesis Induce Differential Vascular Effects. The Role of the Endothelial Glycocalyx
Source: Front Med (Lausanne). 2022 Jun 29;9:889952. doi: 10.3389/fmed.2022.889952 (PMC9277019; doi:10.3389/fmed.2022.889952)

Supplementary Figures

This document provides comprehensive information regarding the effect of HIA 50 and 100 U under control conditions in presence of Phe and ACh in aorta rings. Once HA was removed, tissues were precontracted with Phe and later addition of ACh confirmed the magnitude of the vasodilation and consequent vessel viability (Figure S1).

A preliminary histological analysis was conducted in the isolated rat aorta rings; we aimed to determine if AuNPs could be onto the tissues.

The aortic rings with or without treatment with HIA and AuNPs-CA were suspended in organ baths containing 2 % glutaraldehyde for 2 h, at 4 °C, and then washed out with KH solution. Later, tissues were dehydrated at ambient temperature in various ethanol solutions of 50-100 % for 2 h in each one and finally washed out with KH solution. Finally, all aortic ring samples were dried at ambient temperature for 24 h. Tissues were attached to the sample holder via copper tape, and they were carried in the Scanning Electron Microscopy (SEM) observation and elemental analysis. The percentages of gold atomic were assessed by evaluating the surface chemistry of the tissue samples with X-ray photoelectron spectroscopy, and these percentages were normalized by the area of the tissue samples obtained by Image J software (Version 1.50, National Institute of Health, Bethesda, MD).

Thus, Figure S2A is a representative SEM image of aorta tissue (no AuNPsCA, control), where it was not observed deposition of AuNPs onto the surface of the sample. Figure S2B is a SEM image of aorta tissue treated with 100 µg/mL of AuNPsCA, where deposition of AuNPs onto the surface of the sample was observed. Additionally, the gold (Au^0) concentration (weight) was 19.19% on the tissue.

Supplementary Figure S1. Vascular effects induced by different HIA U, in presence of Phe and ACh. Effects induced by ACh 10 μM on precontracted aortic rings with Phe 2 μM (absence of HIA) (a). Effects induced by HIA b) 50 U in presence of Phe and later addition of ACh and c) 100 U in presence of Phe and later addition of ACh. Results are representative of three independent experiments. Percentage of contraction and relaxation induced by HIA (0, 50 and 100 U) (d) the percentage of tension was based on 100% contraction induced by Phe 2 μM. Values are represented as mean ± SEM (n=3). $^*P < 0.05$, $^{**}P < 0.01$ vs control; and $+P < 0.05$ vs HIA 50 U.

Supplementary Figure S2. SEM-EDX analysis of isolated aorta ring preparation. A) SEM image and EDX analysis of tissue not exposed to AuNPsCA (control), Au (0) was not detected. B) SEM image and EDX analysis of tissue exposed to 100 μg/mL of AuNPsCA, Au (0) was detected in the sample. The instrument used was a JSM 6610-LV (JEOL Company, Peabody, MA) working at an accelerating voltage of 30 kV and coupled with EDX.

Supplementary Figure S1

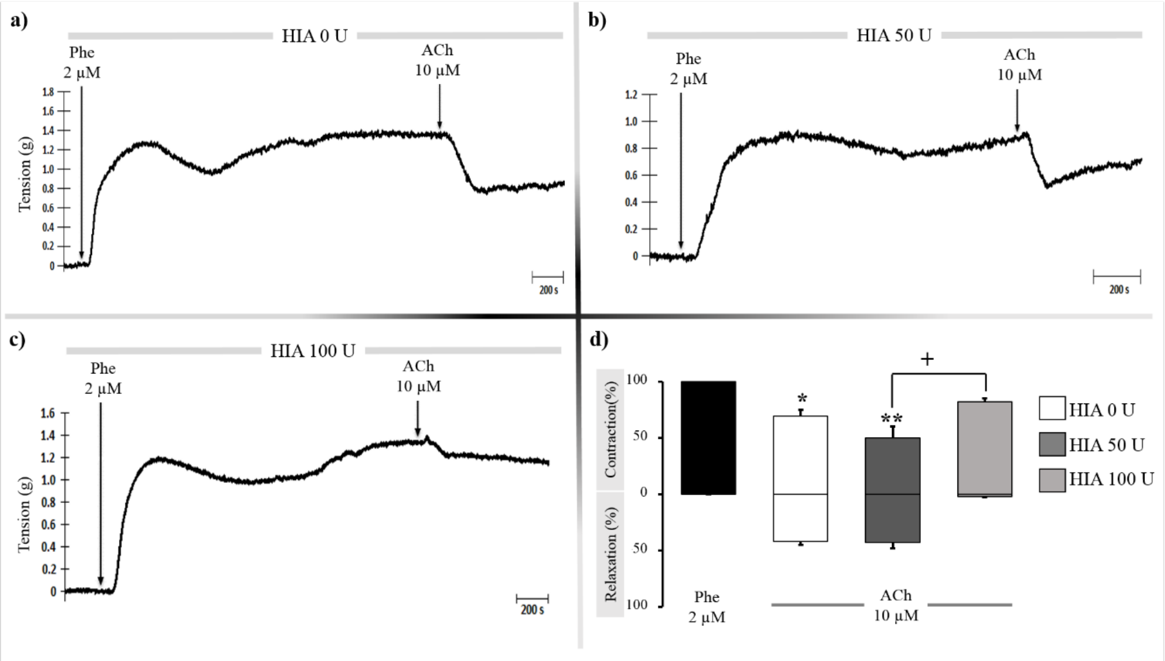

Supplementary Figure S2A

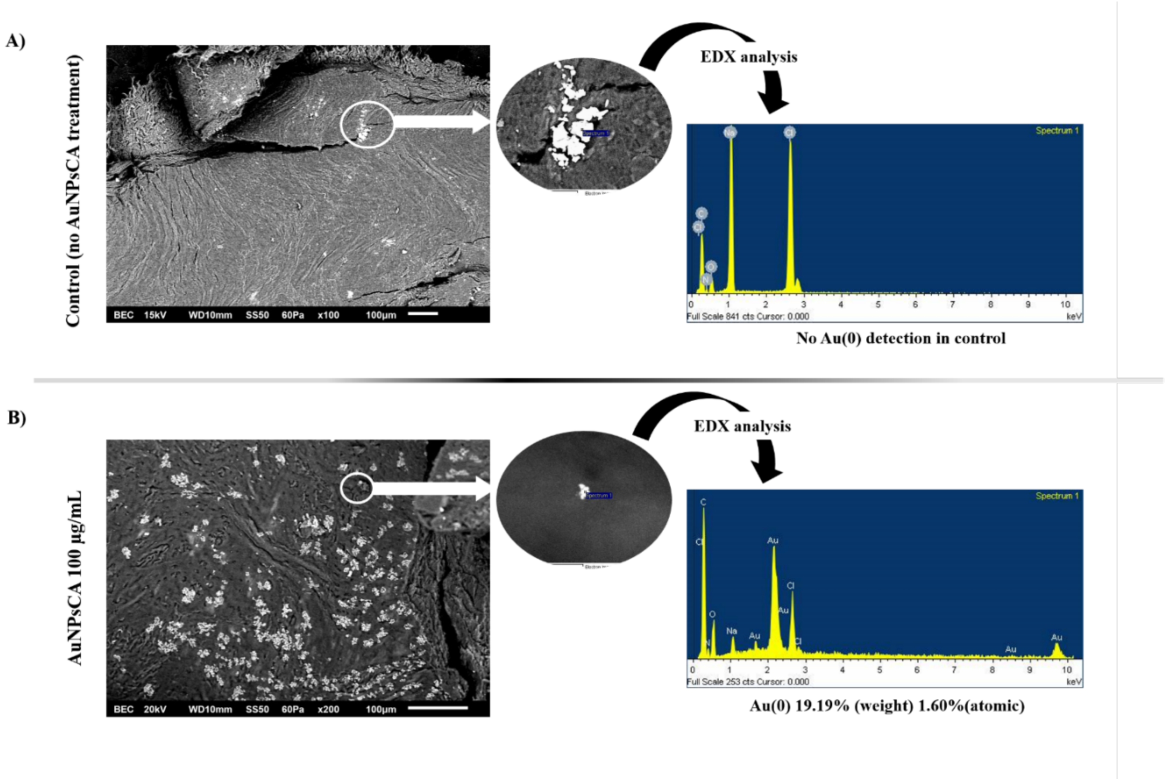

Supplement: Supplementary file 1 [file Data_Sheet_1.pdf]
